# DAPredict: a database for drug action phenotype prediction

**DOI:** 10.1093/database/baad095

**Published:** 2024-01-18

**Authors:** Qingkang Meng, Yiyang Cai, Kun Zhou, Fei Xu, Diwei Huo, Hongbo Xie, Meini Yu, Denan Zhang, Xiujie Chen

**Affiliations:** Department of Pharmacogenomics, College of Bioinformatics Science and Technology, Harbin Medical University, Harbin 150081, China; Department of Pharmacogenomics, College of Bioinformatics Science and Technology, Harbin Medical University, Harbin 150081, China; Department of Pharmacogenomics, College of Bioinformatics Science and Technology, Harbin Medical University, Harbin 150081, China; Department of Pharmacogenomics, College of Bioinformatics Science and Technology, Harbin Medical University, Harbin 150081, China; The Fourth Affiliated Hospital of Harbin Medical University, Harbin 150081, China; Department of Pharmacogenomics, College of Bioinformatics Science and Technology, Harbin Medical University, Harbin 150081, China; Department of Pharmacogenomics, College of Bioinformatics Science and Technology, Harbin Medical University, Harbin 150081, China; Department of Pharmacogenomics, College of Bioinformatics Science and Technology, Harbin Medical University, Harbin 150081, China; Department of Pharmacogenomics, College of Bioinformatics Science and Technology, Harbin Medical University, Harbin 150081, China

## Abstract

The phenotypes of drug action, including therapeutic actions and adverse drug reactions (ADRs), are important indicators for evaluating the druggability of new drugs and repositioning the approved drugs. Here, we provide a user-friendly database, DAPredict (http://bio-bigdata.hrbmu.edu.cn/DAPredict), in which our novel original drug action phenotypes prediction algorithm (Yang,J., Zhang,D., Liu,L. *et al.* (2021) Computational drug repositioning based on the relationships between substructure-indication. *Brief. Bioinformatics*, 22, bbaa348) was embedded. Our algorithm integrates characteristics of chemical genomics and pharmacogenomics, breaking through the limitations that traditional drug development process based on phenotype cannot analyze the mechanism of drug action. Predicting phenotypes of drug action based on the local active structures of drugs and proteins can achieve more innovative drug discovery across drug categories and simultaneously evaluate drug efficacy and safety, rather than traditional one-by-one evaluation. DAPredict contains 305 981 predicted relationships between 1748 approved drugs and 454 ADRs, 83 117 predicted relationships between 1478 approved drugs and 178 Anatomical Therapeutic Chemicals (ATC). More importantly, DAPredict provides an online prediction tool, which researchers can use to predict the action phenotypic spectrum of more than 110 000 000 compounds (including about 168 000 natural products) and corresponding proteins to analyze their potential effect mechanisms. DAPredict can also help researchers obtain the phenotype-corresponding active structures for structural optimization of new drug candidates, making it easier to evaluate the druggability of new drug candidates and develop more innovative drugs across drug categories.

**Database URL:**  http://bio-bigdata.hrbmu.edu.cn/DAPredict/

## Introduction

Drug action phenotypes, also known as drug responses, refer to the sum of all reactions that occur in the organism after medication, including therapeutic effects and adverse drug reactions (ADRs) (1). The goal of drug research and development (R&D) is to discover new drug candidates (NDCs) that exhibit high treatment effects and minimal adverse reactions. To achieve this goal, understanding the spectrum of therapeutic effects and the most likely serious adverse reactions is crucial for evaluating the druggability of new drug candidates and repositioning the approved drugs. However, the current experimental methods in drug discovery are usually based on disease models, and its process is costly, time-consuming, and has a high risk of failure (2, 3), especially the inability to obtain comprehensive action phenotypes, which is a huge challenge for determining the effects on the body of new discovering natural products or new synthesized compounds. While traditional drug phenotype discovery methods are usually referred to the records in ancient medical books or based on folk experience, these can only determine one or several action phenotypes, rather than all action phenotypes, which need to study the disease models of various human organs one by one to determine. Therefore, there is an urgent need to develop calculation methods to break through the above limitations and provide guidance for further experimental research (4).

Current researches only choose chemical-genomic features (drug substructure-protein domain) or pharmacogenomic features (drug adverse reaction-protein domain) and does not integrate chemical-genomic features with pharmacogenomic features to identify the relationship between substructure and action phenotype. This is crucial for predicting drug action phenotype profiles solely based on substructure information of new drug candidates, especially, for natural products.

Most current calculation methods for predicting drug action phenotypes are based on the principle of overall structural similarity of drugs. The basic assumption underlying similarity-based calculations is that structurally similar drugs possess similar functions and, consequently, exhibit similar phenotypic effects. However, these methods have trouble with inconsistencies between structure and function, resulting in what is known as the ‘activity cliff’ phenomenon. This phenomenon implies that the same protein can interact with multiple compounds that are structurally dissimilar or that the same drug can interact with evolutionarily distant proteins (5). As a result, there is an increasing demand for explaining the interaction mechanisms between drugs and target proteins at a higher resolution, leading to a growing focus on drug development models based on local similarity. The active structural groups of drugs and protein domains serve as the basis for drug-target protein interactions. The same active structural group or domain can exist in different compounds and proteins. Developing drugs based on the interaction between active structural groups of drugs and domains of proteins can effectively showcase the local similarity of drugs and broaden the prediction scope of drug-phenotype relationships, ultimately facilitating innovative drug discovery (6).

**Figure 1.**
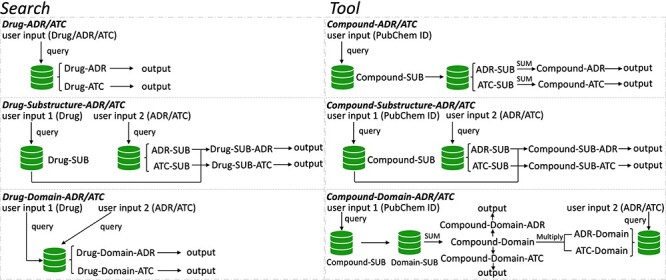
The framework of DAPredict. This figure shows the operating principle of DAPredict, which corresponds to the ‘Search’ and ‘Tool’ sections on the web page respectively. (Abbreviation, SUB: Substructure).

To speed up solving the above problems, we created a novel original drug action phenotypes prediction algorithm (7), which integrated chemical-genomic features and pharmacogenomic features and is based on the local active structures of drugs and proteins. Our method not only achieves the goal of predicting the comprehensive action phenotype of drugs but also enables new drug discovery across drug classes because it is based on local active structures, which greatly expands the space for new drug development and facilitates structural optimization. Finally, we provide DAPredict (http://bio-bigdata.hrbmu.edu.cn/DAPredict), a user-friendly database that embeds our original algorithm for pharmacists to aid in drug discovery (Figure 1).

**Alt text:** A frame diagram showing how the database works.

## Methods

### Data collection and processing

Our database mainly contains the following seven relationship pairs, which are drug-ADR, drug-ATC (here, therapeutic effects represented by the third-level ATCs), compound substructure-ADR, compound substructure-ATC, compound substructure-protein domain, protein domain-ADR and protein domain-ATC. These relationship pairs all come from our original prediction algorithms. Specifically, our prediction algorithm constructed the above seven relationship pairs stored in this database based on drug target data and drug therapeutic effect data from the DrugBank database, 881-bit drug substructure data from the PubChem database and protein domain information from the Pfam database.

For the online prediction tool, we downloaded the compound information in XML format, preprocessed it into compound-substructure relationship pairs and then stored them in the database.

### Full phenotypic profile

Predicted drug action phenotype relationships (including drug-ADR and drug-ATC) are stored in the database, and users can directly retrieve the above-mentioned relationship pairs by inputting the interest drug or phenotype to explore the full phenotypic profile of the interest drug.

### Mechanism of drug action

Drugs usually play a role in the body by binding to target proteins. The most crucial binding site is the domain of the target protein. Therefore, by analyzing the domain of the target protein, researchers can further explore the mechanism by which the drug exerts its effect or produces adverse reactions.

Based on the compound-substructure relationships from the PubChem database and the substructure-domain and domain-ADR or ATC relationships from our algorithm, drug-domain-ADR or ATC relationship data were finally constructed and stored in the database. The relationship score is calculated as follows:


$$Score = {{\Sigma }}_{j = 1}^{j = n}Co{r_{sub\,i\, - \,domain\,j}}*Co{r_{domain\,j\, - \,ADR\,or\,ATC}}$$

$Co{r_{sub{\mathrm{\,}}i{\mathrm{\,}} - {\mathrm{\,}}domain{\mathrm{\,}}j}}$ represents the relationship score between substructure *i* of a given drug and domain *j*, $Co{r_{domain{\mathrm{\,}}j{\mathrm{\,}} - {\mathrm{\,}}ADR{\mathrm{\,or\,}}ATC}}$ represents the relationship score between domain *j* and a given ADR or ATC, where *n* represents the number of intersection domains in the substructure-domain and domain-ADR or ATC relationships.

### Drug phenotype-associated substructures

The phenotype-related substructure is the most important information in structural optimization of drug development to eliminate serious adverse drug reactions (SADR). Here, the database provides researchers with valuable drug action phenotype-related substructure information based on compound-substructure relationships from the PubChem database and substructure-ADR or ATC relationships from our algorithm. The relationship score is calculated as follows:


$$Score = Co{r_{sub\,i\, - \,ADR\,or\,ATC}}$$

$Co{r_{sub\,i\, - \,ADR\ \,or\ \,ATC}}$ represents the relationship score between substructure *i* of a given drug and a given ADR or ATC.

### Online prediction tool

In addition to the search function of existing data, our database also provides an online prediction tool, which can apply our prediction algorithm to almost all compounds.

Specifically, for predicting the compound-ADR or ATC relationships, the calculation formula is as follows:


$$Score = {{\Sigma }}_{i = 1}^{i = n}Co{r_{sub\,i\, - \,ADR\,j\,or\,ATC\,j\,}}$$

$Co{r_{sub{\mathrm{\,}}i{\mathrm{\,}} - {\mathrm{\,}}ADR{\mathrm{\,}}j{\mathrm{\,}}or{\mathrm{\,}}ATC{\mathrm{\,}}j}}$ represents the relationship score between substructure *i* and ADR j or ATC *j*, where *n* represents the number of substructures of a given compound.

For predicting compound-domain-ADR or ATC relationships, the calculation formula is as follows:


$$Score = {{\Sigma }}_{j = 1}^{j = n}Co{r_{sub\,i\, - \,domain\,j}}*Co{r_{domain\,j\, - \,ADR\,or\,ATC}}$$

$Co{r_{sub{\mathrm{\,}}i{\mathrm{\,}} - {\mathrm{\,}}domain{\mathrm{\,}}j}}$ represents the relationship score between substructure *i* of a given compound and domain *j*, $Co{r_{domain{\mathrm{\,}}j{\mathrm{\,}} - {\mathrm{\,}}ADR{\mathrm{\,or\,}}ATC}}$ represents the relationship score between domain *j* and a given ADR or ATC, where *n* represents the number of intersection domains in the substructure-domain and domain-ADR or ATC relationships. But unlike the search function, these calculations are performed online in real time.

For predicting compound-substructure-ADR or ATC relationships, the calculation formula is as follows:


$$Score = Co{r_{sub\,i\, - \,ADR\,or\,ATC}}$$

$Co{r_{sub\,i\, - \,ADR\,or\,ATC}}$ represents the relationship score between substructure *i* of a given compound and a given ADR or ATC.

### Implement

DAPredict has been developed with Django version 3.2 and Python version 3.7.

## Results

### Data in the DAPredict database

DAPredict contains a total of 1748 approved drugs, 454 ADRs, 178 ATCs and 111 428 061 compounds. Among them, 178 ATCs covered all 14 first-level ATCs, and compounds covered 168 360 natural products. All relationship pairs are counted as shown in Table 1; there are 305 981 drug-ADR relationships and 83 117 drug-ATC relationships. A total of 5808 pairs of substructure-ADR relationships, 1748 pairs of substructure-ATC relationships, 33 334 pairs of substructure-domain relationships, 30 874 pairs of domain-ADR relationships and 2788 pairs of domain-ATC relationships came from our prediction algorithm.

**Table 1.** Data in DAPredict. Data: This column represents the type of stored data, including known information obtained from existing databases and relationship pairs predicted by our original algorithm. Source: This column represents the source of stored data. Number: This column represents the number of entries of stored data. Description: This column represents the detailed description of stored data. (Abbreviation, SUB: Substructure)

Data	Source	Number	Description
Approved drug	DrugBank	1748	Approved drugs for predicting the action phenotype.
ADR	SIDER	454	Adverse reactions to be predicted.
ATC	DrugBank	178	Drug therapy effects to be predicted.
Compound	PubChem	111 428 061	Compounds for predicting the action phenotype online.
Drug-ADR	Original algorithm	305 981	Drug-ADR predicted relationships.
Drug-ATC	Original algorithm	83 117	Drug-ATC predicted relationships.
SUB-ADR	Original algorithm	5808	Substructure-ADR predicted relationships.
SUB-ATC	Original algorithm	1748	Substructure-ATC predicted relationships.
SUB-Domain	Original algorithm	33 334	Substructure-domain predicted relationships.
Domain-ADR	Original algorithm	30 874	Domain-ADR predicted relationships.
Domain-ATC	Original algorithm	2788	Domain-ATC predicted relationships.

### Relationship score grade

To intuitively quantify the degree of relationship scores, all relationship scores are used as the background, and the scores are divided into five levels by quintiles, namely lowest, low, medium, high and highest. The division thresholds for each relationship are shown in Table 2.

**Table 2.** Score grade threshold. Relationship: This column shows the names of relationship pairs. Grade: This column represents the five levels of correlation, namely highest, high, medium, low and lowest. Threshold: This column shows the correlation range of each level. (Abbreviation, SUB: Substructure)

Relationship	Grade	Threshold
Drug-ADR	Highest	(0.000630035,+∞)
Drug-ADR	High	(0.0000172,0.000630035]
Drug-ADR	Medium	(1.64E-10,0.0000172]
Drug-ADR	Low	(3.56E-21,1.64E-10]
Drug-ADR	Lowest	(0,3.56E-21]
Drug-ATC	Highest	(0.00124725,+∞)
Drug-ATC	High	(0.00000675,0.00124725]
Drug-ATC	Medium	(7.45E-10,0.00000675]
Drug-ATC	Low	(3.28E-22,7.45E-10]
Drug-ATC	Lowest	(0,3.28E-22]
Drug-SUB-ADR	Highest	(0.1976036,+∞)
Drug-SUB-ADR	High	(0.00054703,0.1976036]
Drug-SUB-ADR	Medium	(3.480196e-16,0.00054703]
Drug-SUB-ADR	Low	(5.73442e-26,3.480196e-16]
Drug-SUB-ADR	Lowest	(0,5.73442e-26]
Drug-SUB-ATC	Highest	(0.749394,+∞)
Drug-SUB-ATC	High	(0.296104,0.749394]
Drug-SUB-ATC	Medium	(0.000727606,0.296104]
Drug-SUB-ATC	Low	(3.13E-13,0.000727606]
Drug-SUB-ATC	Lowest	(0,3.13E-13]
Drug-Domain-ADR	Highest	(18.3027,+∞)
Drug-Domain-ADR	High	(10.7562,18.3027]
Drug-Domain-ADR	Medium	(6.08470,10.7562]
Drug-Domain-ADR	Low	(2.63413,6.08470]
Drug-Domain-ADR	Lowest	(0,2.63413]
Drug-Domain-ATC	Highest	(24.3574,+∞)
Drug-Domain-ATC	High	(15.7606,24.3574]
Drug-Domain-ATC	Medium	(10.2282,15.7606]
Drug-Domain-ATC	Low	(4.94737,10.2282]
Drug-Domain-ATC	Lowest	(0,4.94737]

Since the prediction of compounds is performed online, it is impossible to obtain the relationship score between all compounds and phenotypes in advance to construct the background, and the approved drugs that have been predicted in advance are representative of all compounds, so the score thresholds of the online prediction were set consistent with the approved drugs.

### Web interface and visualization

The web page is divided into five sections, namely home, search, tool, download and help. The home section shows the introduction of DAPredict, and the search section provides users with the direct search function of the pre-predicted full action phenotype spectrum, potential action domain and action phenotype-related substructures of 1748 approved drugs. The tool section provides users with online real-time prediction functions of the full action phenotype spectrum, potential action domain and action phenotype-related substructures of more than 110 000 000 compounds included in the PubChem database. The download section provides users with a wealth of downloadable resources, including the pre-predicted full action phenotype relationship data of 1748 approved drugs, and the relationship data between substructure and ADR, ATC and target protein domain. In addition, DAPredict also includes drug-target relationship data from the DrugBank database and target-domain relationship data from the Pfam database. The help section describes browser compatibility and specific usage tutorial of DAPredict.

**Figure 2.**
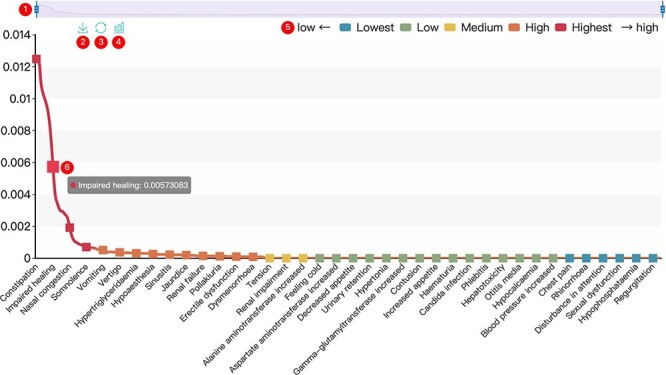
Interactive graph. ① Sliding window: Users can drag the handles at both ends to adjust the size of the sliding window. Drag the sliding window to further explore a specific small range of data. ② Save: Users can save the current graph. ③ Restore: Users can restore the graph to its original form. ④ Bar plot: Users can click this button to switch the default line chart to a bar chart. When the score is very small, it is recommended to view the bar chart. ⑤ Rank: The predicted relevance score is divided into five grades (see the search and tool sections for details), and users can click the label to filter the results by rank attribute. ⑥ Interactive nodes: Users can hover over nodes to view details.

**Figure 3.**
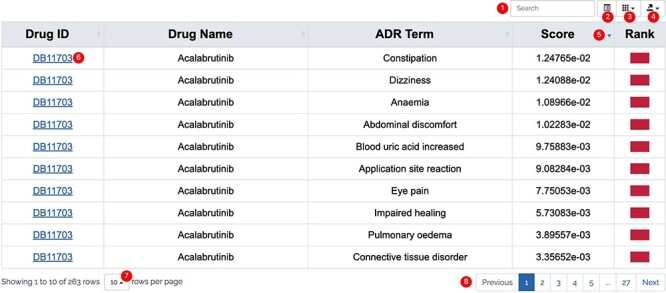
Interactive table. ① Search: search within the form is available to further filter valuable results. ② Format conversion: users can switch the present format of the result form. ③ Column display: users can customize the displayed columns. ④ Export: users can export the result on the current page in CSV or Excel formats (to download all results, please select display number as all.). ⑤ Arrange: users can sort the form in ascending or descending order based on a column. ⑥ Related link: hyperlink is underlined in blue, and users can click to go to the relevant page. ⑦ Display number: the number of results displayed on the current page. ⑧ Page: users can turn pages here.

All results in DAPredict are presented in interactive graphs and tables. The forms of interactive graphs include line plots (default) and bar plots (Figure 2). The grade of the relationship score is distinguished by different colors and can be displayed selectively by users in graphs. Due to the limited space on the webpage, we have set a sliding window function for interactive graphs, and users can view the results within the sliding window in more detail by dragging the sliding window. The interactive table provides users with column sorting and a secondary search function by which users can further filter keywords in the search results (Figure 3). Clickable hyperlinks in the table are underlined, and users can easily jump to related sites. Both graphs and tables are available for download in DAPredict.

**Alt text:** This figure demonstrates the use of interactive graphs in the database.

**Alt text:** This figure demonstrates the use of interactive tables in the database.

## Discussion

We have developed a comprehensive and easy-to-use drug action phenotype search and prediction platform based on our original prediction algorithm, which includes not only the pre-predicted full action phenotype profile of 1748 approved drugs but also the ability to predict more than 110 000 000 known compounds online. It is valuable to reposition approved drugs with reference to the predicted full action phenotype profile, which would save a lot of manpower, resources, finances and time compared to de novo drug development. In addition, the screening of lead compounds is one of the most important steps in the development of new drugs. Predicting and evaluating the action phenotype of known compounds can provide a reference value for the screening of lead compounds, which also includes natural products that are less studied at present. More importantly, DAPredict can also provide researchers with information on the mechanism of action of a drug or compound and substructure information related to the action phenotype, which is of great significance in druggability evaluation and structural optimization in the development of new drugs.

At present, the number of known compounds is constantly increasing, so our database needs to be constantly updated. In addition, the predicted results may still need to be checked manually. This would be a time-consuming process because of the huge number of compounds. We hope that DAPredict can provide a convenient and valuable data resource for drug developers and pharmacogenomics researchers.

## Data Availability

All data in DAPredict are stored in the download section, and users can download it on demand.

## Code availability

No custom code was used. Software tools used for processing are mentioned in the Methods section.
